# ROS/TRPA1/CGRP signaling mediates cortical spreading depression

**DOI:** 10.1186/s10194-019-0978-z

**Published:** 2019-03-06

**Authors:** Liwen Jiang, Dongqing Ma, Blair D. Grubb, Minyan Wang

**Affiliations:** 1 Centre for Neuroscience, Xi’an Jiaotong-Liverpool University, Suzhou, China; 2 Department of Biological Sciences, Xi’an Jiaotong-Liverpool University, 111 Renái Road, Suzhou, 215123 People’s Republic of China; 3 Institute of Translational Medicine, University of Liverpool, Crown Street, Liverpool, UK

**Keywords:** Migraine, Cortical spreading depression, Transient receptor potential ankyrin a 1, Reactive oxygen species, Calcitonin gene-related peptide

## Abstract

**Objectives:**

The transient receptor potential ankyrin A 1 (TRPA1) channel and calcitonin gene-related peptide (CGRP) are targets for migraine prophylaxis. This study aimed to understand their mechanisms in migraine by investigating the role of TRPA1 in cortical spreading depression (CSD) in vivo and exploring how reactive oxygen species (ROS)/TRPA1/CGRP interplay in regulating cortical susceptibility to CSD.

**Methods:**

Immunohistochemistry was used for detecting TRPA1 expression. CSD was induced by K^+^ on the cerebral cortex, monitored using electrophysiology in rats, and intrinsic optical imaging in mouse brain slices, respectively. Drugs were perfused into contralateral ventricle of rats. Lipid peroxidation (malondialdehyde, MDA) analysis was used for indicating ROS level.

**Results:**

TRPA1 was expressed in cortical neurons and astrocytes of rats and mice. TRPA1 deactivation by an anti-TRPA1 antibody reduced cortical susceptibility to CSD in rats and decreased ipsilateral MDA level induced by CSD. In mouse brain slices, H_2_O_2_ facilitated submaximal CSD induction, which disappeared by the antioxidant, tempol and the TRPA1 antagonist, A-967079; Consistently, TRPA1 activation reversed prolonged CSD latency and reduced magnitude by the antioxidant. Further, blockade of CGRP prolonged CSD latency, which was reversed by H_2_O_2_ and the TRPA1 agonist, allyl-isothiocyanate, respectively.

**Conclusions:**

ROS/TRPA1/CGRP signaling plays a critical role in regulating cortical susceptibility to CSD. Inhibition ROS and deactivation of TRPA1 channels may have therapeutic benefits in preventing stress-triggered migraine via CGRP.

## Article highlights

- Deactivation of TRPA1 reduces the likelihood for CSD occurrence in rats, which coincides with lower level of lipid peroxidation induced by CSD.
- ROS/TRPA1/CGRP signaling plays a critical role in regulating cortical susceptibility to CSD, which is of central mechanism.
- Reduction of ROS production and blockade of TRPA1 may have therapeutic benefits in preventing stress-triggered migraine.

## Introduction

Migraine is a complex disabling neurovascular disorder, which has unmet therapeutic need due to its complicated mechanism that is not yet fully understood. Cortical spreading depression (CSD) is a key pathophysiological basis of migraine aura [1–3]. The importance of CSD in migraine pathogenesis is supported by that inhibition of calcitonin gene-related peptide (CGRP) and its receptor not only prevents chronic and episodic migraine [1, 4], but also suppresses cortical susceptibility to CSD [5, 6]. One common factor for almost all migraine triggers is reactive oxygen species (ROS) [7]. Increased ROS is associated with migraine [8, 9] but also with CSD in the trigeminal nociceptive system [10]. This is supported by that the antioxidant, tempol, strongly reduces the CSD occurrence in vivo [11]; while the lowered-CSD threshold level is considered as stress-induced susceptibility to migraine [12]. Given that CSD activates trigeminovascular neurons and meningeal nociceptors [13, 14], molecules in the oxidative stress pathway that can modulate CSD are likely to be important targets in stress-related migraine.

One such emerging drug target for migraine prevention involves the transient receptor potential ankyrin type 1 (TRPA1). TRPA1 is sensitive to oxidative stress and is a type of nonselective transmembrane cation channel with 14–18 ankyrin repeats in its N-terminus depending on species. This channel is widely expressed in rodent cerebral cortex [15, 16], hippocampus [16–18] and trigeminal nociceptive system [19], yet TRPA1 distribution in cortical neurons or glias is not known. TRPA1 deactivation has been recently reported to counteract nitroglycerin-induced hyperalgesia [20]; whilst the channel activation mediates allodynia induced by glyceryl trinitrate in mice [21]. Coincidently, we recently show that TRPA1 plays a key role in mediating CSD in the mouse brain slice [5], suggesting TRPA1 as a drug target for preventing migraine aura. However, whether deactivation of TRPA1 reduces cortical susceptibility to CSD in vivo where vascular components and immune system are preserved is still not clear.

One key downstream signal of TRPA1 in migraine may involve CGRP as previously studies reported that TRPA1 activation mediates ROS-induced CGRP release in the rat trigeminal ganglion [10] and in the dura mater [22]; whilst exogenous CGRP is able to reverse the inhibitory action on CSD under TRPA1 deactivation in vitro [5]. Yet, how these signals interact in regulating cortical susceptibility to CSD is not fully understood. In the present study, we investigated the role of TRPA1 in CSD in vivo and further explored functional relationship among ROS/TRPA1/CGRP in regulating cortical susceptibility to CSD. We demonstrated that deactivation of TRPA1 reduces not only cortical susceptibility to CSD in rats but also attenuated CSD-induced cortical lipid peroxidation. Our data also showed that the key role of TRPA1 in regulating cortical susceptibility to CSD is functionally related to ROS and CGRP and their actions are of central mechanism. We propose that ROS triggers TRPA1 activation and CGRP production, which create a positive feedback loop in regulating cortical susceptibility to CSD. In which way, ROS could facilitate CSD propagation for the subsequent development of migraine.

## Experimental procedures

### Animal use

A total of 30 male Sprague Dawley rats (253.8 ± 40.9 g) and 36 male C57BL6 mice (21.2 ± 2.7 g, mean ± SD) were purchased from Shanghai SLAC Laboratory Animal Corporation Ltd. The sample size of animals was estimated based on previous studies on CSD susceptibility [5, 23]. Animals were housed in the experimental animal Centre of Soochow University under the agreement with Xi’an Jiaotong-Liverpool University (XJTLU) for at least one-week before use. All animal procedures were approved by the Ethic Review Panel of Soochow University and performed in accordance with the relevant national and provincial guidelines.

### Immunohistochemistry

TRPA1 expression in cortical neurons and astrocytes of rats and mice was detected using immunohistochemistry (IHC). Rats (*n* = 3) and mice (*n* = 3) were anaesthetized with isoflurane (5% in O2:N2O (1:2)), followed by cardiac perfusion with phosphate buffered saline (PBS, 09–8912-100, Medicago), fixation with 4% paraformaldehyde (PFA) overnight and cryo-protected in 10% to 30% sucrose solution overnight orderly for dehydration. The brain was embedded in Tissue-Tek® O.C.T Compound (Sakura, Finetek, Torrance, CA, USA). Coronal sections (10 μm) between 1 and 1.5 mm posterior to bregma, which contains somatosensory and motor cortex regions, were prepared using a cryostat (CM1950, Leica, China).

Brain slices were washed 3 × 15 min in PBS, and then incubated with a blocking solution (10% normal goat serum in PBS with 0.1% Tween 20) for 2 h at room temperature. Double staining was performed with anti-TRPA1 antibody (1:200, Alomone Labs, ACC-0037) and either anti-NeuN antibody (1:1000, MAB377) indicating neurons or anti-glial fibrillary acidic protein (GFAP) antibody (1:200, CST3670) indicating glial cells. Briefly, after incubation with the primary antibodies overnight, cortical sections were brought to room temperature, rinsed in PBS and exposed to the secondary antibodies (Alexa Fluor® 488 Goat Anti-Mouse IgG (H + L) Antibody, 1:500, Invitrogen, A-11029; Alexa Fluor® 568 Goat Anti-Rabbit IgG (H + L) antibody, 1:500, Invitrogen, A-11036) in blocking solution for 1 h at room temperature. The specimens were subsequently washed with PBS, and incubated with 0.2% of 4′,6-diamidino-2-phenylindole (DAPI, Sigma, D8417) for 5 min. After washed, specimens were mounted with SlowFade® Gold Antifade Mountant (Invitrogen, S36936). TRPA1 localization in the cortical neurons and astrocytes was identified using a fluorescence microscope (Nikon, ECLIPSE Ni-U or confocal microscope, LSM880, Zeiss). Immuno-negative control was performed by staining of anti-IgG antibody in the rat and mice cortices.

### In vivo surgery for antibody perfusion and CSD induction in rats

The in vivo surgery for antibody perfusion into the intracerebral ventricle (*i.c.v*) and CSD induction in rats was under anesthesia using isoflurane as described previously [23, 24]. Total 29 rats were used. Three burr holes were drilled in the parietal bone of each rat. One burr hole on the left side was drilled using a dental drill (D-3000, Otto Muss). This hole was used for implanting a stainless steel cannula (0.38 mm i.d, RWD Life Science) into the *i.c.v.* (3.5 mm deep from the cortical surface) for the antibody perfusion at 1 μl/minute, followed by rat recovery. The other two burr holes were drilled 4 days later on the right side for CSD induction with dura intact (1 mm, i.d, coordinates: 5 mm posterior and 2 mm lateral to bregma) and CSD recording (0.8 mm, i.d, coordinates: 3 mm anterior and 2 mm lateral to bregma). A reference electrode was positioned under the scalp. The depth of anesthesia was monitored and adjusted according to the electroencephalogram (EEG) signal, breathing and body reflex.

On day 4, multiple CSD waves were induced by continuous perfusion of 2 M KCl for 30 min after 1-h tissue recovery post the surgery. EEG and direct current (DC) signals were first amplified with a high-impedance input, AC/DC pre-amplifier (NL834, Digitimer Ltd., UK). The alternating current component in the 1–30 Hz window provided the EEG (overall × 5000 amplification) and the 0–30 Hz window provided the extracellular DC potential (overall × 250 amplification) at sampling rate of 10 HZ. The recorded EEG variables were monitored by a digital oscilloscope (DS1000B, RIGOL, China). All the signals were continuously recorded and digitized by Labview 11.0 (National Instruments). After CSD recording in vivo, rats were sacrificed immediately and cerebral cortices were dissected, snap frozen for subsequent lipid peroxidation analysis.

### In vivo experimental design

Our previous data show that deactivation of TRPA1 channel either abolishes or suppresses CSD in vitro [5]. In this study, we investigated whether anti-TRPA1 antibody perfused into the *i.c.v.* could reduce cortical susceptibility to CSD in rats. Three groups were designed: (i) pretreatment of the anti-TRPA1 antibody with a total 0.8 μg (Alomone Labs, *n* = 10) 4 days before CSD induction. (ii, iii) Unconjugated rabbit IgG (H + L) (Sangon, D110502, *n* = 8) with a total 0.8 μg being applied for both the CSD group and the sham group as controls (*n* = 7 in each group).

### Lipid peroxidation analysis

We examined whether CSD could increase lipd peroxidation in the rat cerebral cortex; and whether this increase induced by CSD could be prevented after pretreatment of the anti-TRPA1 antibody. To address these, Ipsilateral cerebral cortices of rats in both the anti-IgG and anti-TRPA1 antibodies groups were homogenized in the cold lysis buffer (Beyotime, P0013) with 1X protease inhibitor (Roche) on ice, centrifuged for 10 min at 13000 xg at 4 °C. Total protein levels in the supernatants were determined using the Bicinchoninic Acid Kit (Beyotime, P0010). Malondialdehyde (MDA) levels of these samples indicating the degree of lipid peroxidation were measured at 532 nm using the MDA kit (Beyotime, S0131).

### In vitro CSD induction and imaging of mouse brain slices

Given that deactivation of TRPA1-reduced cortical susceptibility to CSD involves CGRP [5] and CGRP is a downstream product of ROS [10], we investigated whether ROS could facilitate CSD induction and how TRPA1 would interplay with ROS and CGRP in modulating cortical susceptibility to CSD. Preparation of mouse brain slices and intrinsic optical imaging was as described in details previously [5]. Briefly, coronal sections (400 μm) were prepared and perfused with Kreb’s medium at a rate of 3 ml/minute. CSD was induced in the somatosensory region of the brain slice by ejection of KCl. For each KCl application, changes in the reflected intrinsic optical signal (IOS) in the cortical slice were recorded for 15 min as reported earlier [5].

### In vitro experimental design

Two series of experiments were designed in the mouse slice. In order to minimize the animal use, two ways were considered: (i) at least 2 brain slices per mouse were used on the same day and they were assigned to different experimental groups; (ii) Where applicable, relevant data using the same protocol in Series 1 and 2 below as in our recent publication [5] were adopted for comparison (also see Figs. 5 and 6 legends).

*Series 1:* We first examined whether exogenous ROS could facilitate CSD induction in the mouse brain slice and whether this amplification was counteracted by ROS inhibition. Submaximal CSD was induced by KCl at 50 mM as described previously [5] in order to uncover a possible ROS-induced amplification in this phenomenon; The following four groups were designed: (i) Kreb’s (*n* = 6) as control; (ii, iii) 50 μM of the ROS activator, H_2_O_2_ (Sinopharm Chemical Reagent Ltd., 10,011,218) in the absence or presence of 3 mM of tempol (Aladin, K1520232, *n* = 7 in each group), a ROS inhibitor that suppresses CSD in hippocampus slices [11]; (iv) 3 mM of tempol alone (*n* = 7).

We then investigated whether there was a functional link between ROS and TRPA1 channel activation in regulating submaximal CSD, and whether this interaction would be bidirectional. To test this, another two groups were designed: (v) 3 mM of tempol in the presence of 15 μM of the TRPA1 activator, umbellulone (UMB, Sigma-Aldrich, 083m4714V, *n* = 6); (vi) whether facilitation of submaximal CSD by 50 μM of H_2_O_2_ could be reversed by TRPA1 deactivation was also examined by co-application with the TRPA1 antagonist, 1 μM of A967079 (Tocris, Bristol, UK, 4716, *n* = 6).

In this series, two submaximal CSD episodes were elicited in each experiment with a 45-mintue interval for brain slice recovery. The drug or vehicle was perfused 45 min prior to the 2nd submaximal CSD induction for 1 h, except that H_2_O_2_ was pre-incubated with the slice for 30 min. In order to minimize the animal use, data in Kreb’s control group were adopted and transformed from that in Fig. 4 in our previous paper [5].

*Series 2:* We then investigated whether TRPA1 activation or exogenous ROS could reverse the reduced cortical susceptibility to CSD by blockade of CGRP. The following four groups were designed: (x, xi, xii) anti-CGRP antibody (CST, 14959S, *n* = 6) at 0.4 μM in the absence or presence of either 50 μM of the TRPA1 activator, allyl isothiocyanate (AITC, Sigma-Aldrich, 36,682, *n* = 6) or 50 μM of H_2_O_2_ (*n* = 6); (xiii) the anti-IgG antibody (Sangon, D110502, *n* = 6) at 0.025 μM was as the control.

In this series, two CSD episodes were induced by 260 mM KCl in each experiment with a 2-h interval. The antibody was pre-incubated with the brain slice for 1 h starting as soon as the 1st CSD recording was completed for sufficient antibody-antigen binding. AITC or H_2_O_2_ was perfused for 45 min prior to the 2nd CSD induction for 1 h. In order to minimize the animal use, data in anti-IgG antibody control group and the anti-CGRP antibody were adopted and transformed from that in Fig. 5 in our recent paper [5].

### Intrinsic optical image analysis

The image analysis was carried out as described previously [5]. For all the image sequences, the same area of interest (AOI) parallel to each CSD wave was selected and delineated manually in layers 4–6 of the somatosensory cortex that were distant from the site of KCl application. For each picture within the sequence, gray levels of the pixel constituting the AOI were corrected by subtracting the respective dark background. The recorded IOS that was synchronous with sudden cellular depolarization, characterizes the excitation phase of CSD. The average gray level within each AOI was plotted against time to provide a CSD wave.

### Data presentation and statistical analysis

For the in vivo data presentation, electrophysiological data on CSD was quantified using Labview program as described previously [14]. Three parameters were defined to reflect cortical susceptibility to CSD: (i) CSD number in each episode. (ii) CSD latency (minute), i.e., the time difference between the start of KCl application and the starting point of the rising phase of the first CSD wave. (iii) Area under the curve (AUC, mV × minute) of each CSD wave was used to reflect CSD magnitude. The magnitude average in each CSD episode was used for data comparison.

For the image analysis, CSD was quantified as reported previously [22]. Latency (second) was calculated by the time interval between the starting point of KCl ejection and CSD elicitation. AUC was calculated by gray levels × time period. In order to eliminate variations of AOI chosen in each individual experiment *in series 2*, CSD latency within each different test were given in the second CSD episode relative to that of the first CSD wave (i.e. initial control). CSD magnitude within each different test were also given in the second CSD episode relative to that of the first CSD wave (i.e. initial control).

For data analysis, the abnormal distribution test using Shapiro-Wilk was confirmed using Prism software. All values for the above in vivo and in vitro experiments were given in median (range). The Mann-Whitney U test (one-tailed) was used for comparing CSD data of two independent groups for significance and correlation analysis (2-tailed) between MDA level (μmol/mg tissue) and each CSD parameter. Significant differences are shown as *p < 0.05, ** *p* < 0.01 and *** *p* < 0.001.

## Results

### TRPA1 was expressed in neurons and astrocytes of cerebral cortices of rats and mice

**Fig. 1:**
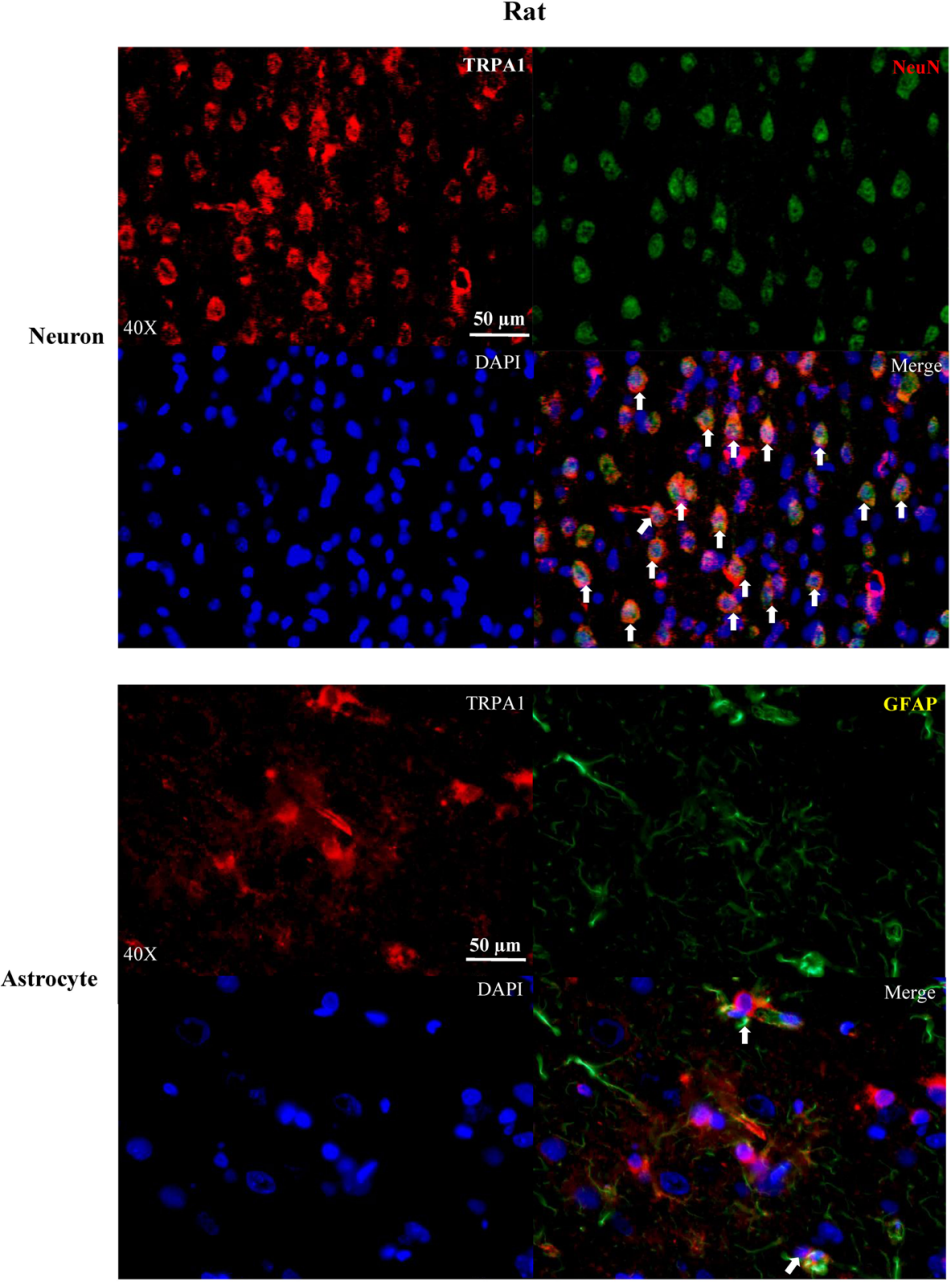
Representative images showing immunohistochemistry detection of TRPA1 expression in neurons and astrocytes of rat cerebral cortex. Double immune-labelling showed TRPA1 expressed in cerebral cortical neurons (upper panel, white arrows) and astrocytes (lower panel, white arrows). DAPI staining indicates nucleus is shown in blue; TRPA1 in red, NeuN indicates neurons or GFAP indicates glial cells shown in green

**Fig. 2:**
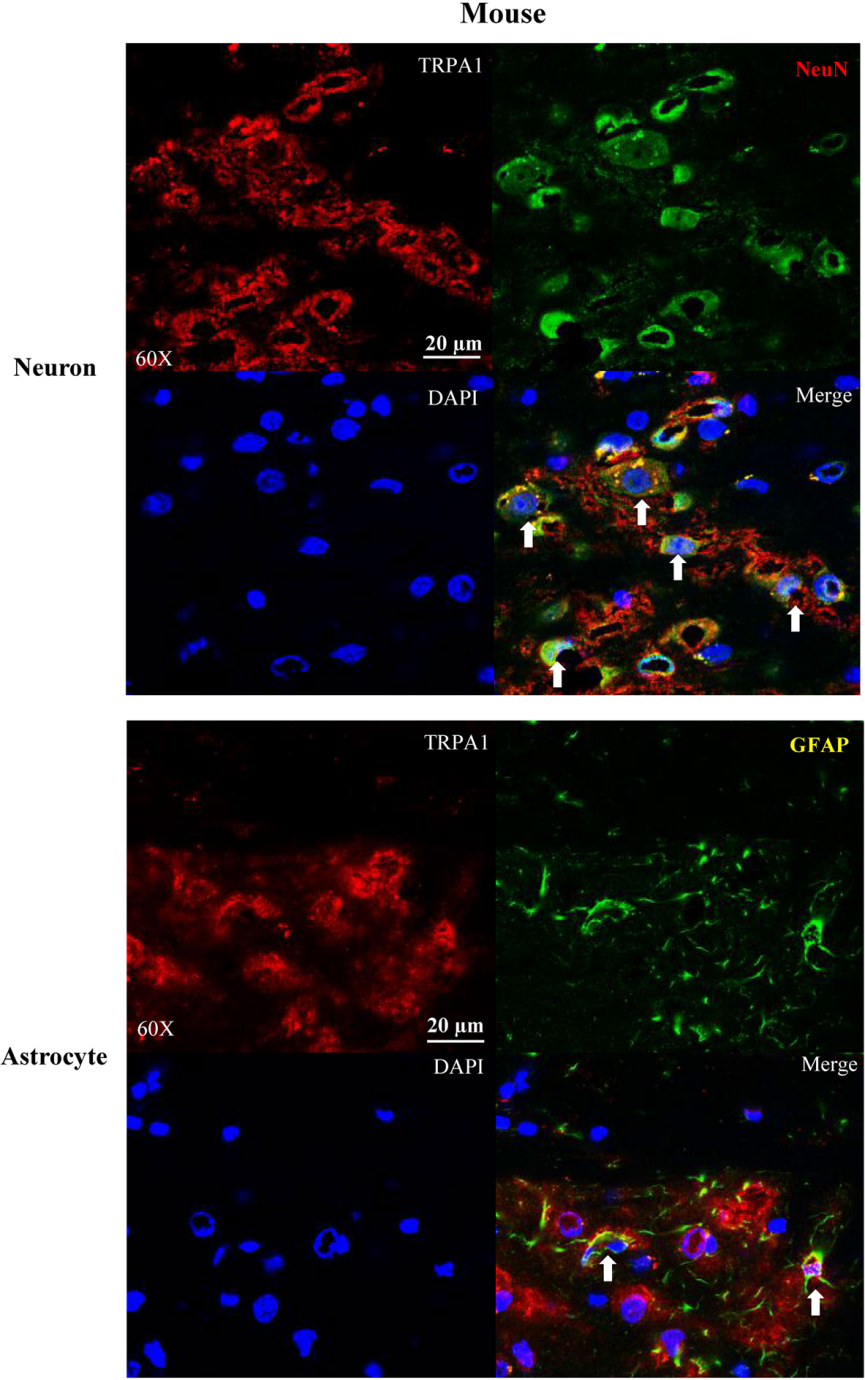
Representative images showing immunohistochemistry detection of TRPA1 expression in neurons and astrocytes of mouse cerebral cortex. Double immune-labelling showed TRPA1 expressed in cerebral cortical neurons (upper panel, white arrows) and astrocytes (lower panel, white arrows). DAPI staining indicates nucleus is shown in blue; TRPA1 in red, NeuN indicates neurons or GFAP indicates glial cells shown in green

TRPA1 was previously reported to be expressed in the cerebral cortices of rats [16]. In the present study, TRPA1 expression was observed in both neurons and astrocytes of rat cerebral cortices (*n* = 3), to a larger extent, in cortical neurons (Fig. 1, upper panel). Similarly, expression of TRPA1 was also observed in neurons and astrocytes of the mouse cerebral cortex (Fig. 2, upper panel, *n* = 3).

### TRPA1 channel deactivation suppressed CSD in rats

**Fig. 3:**
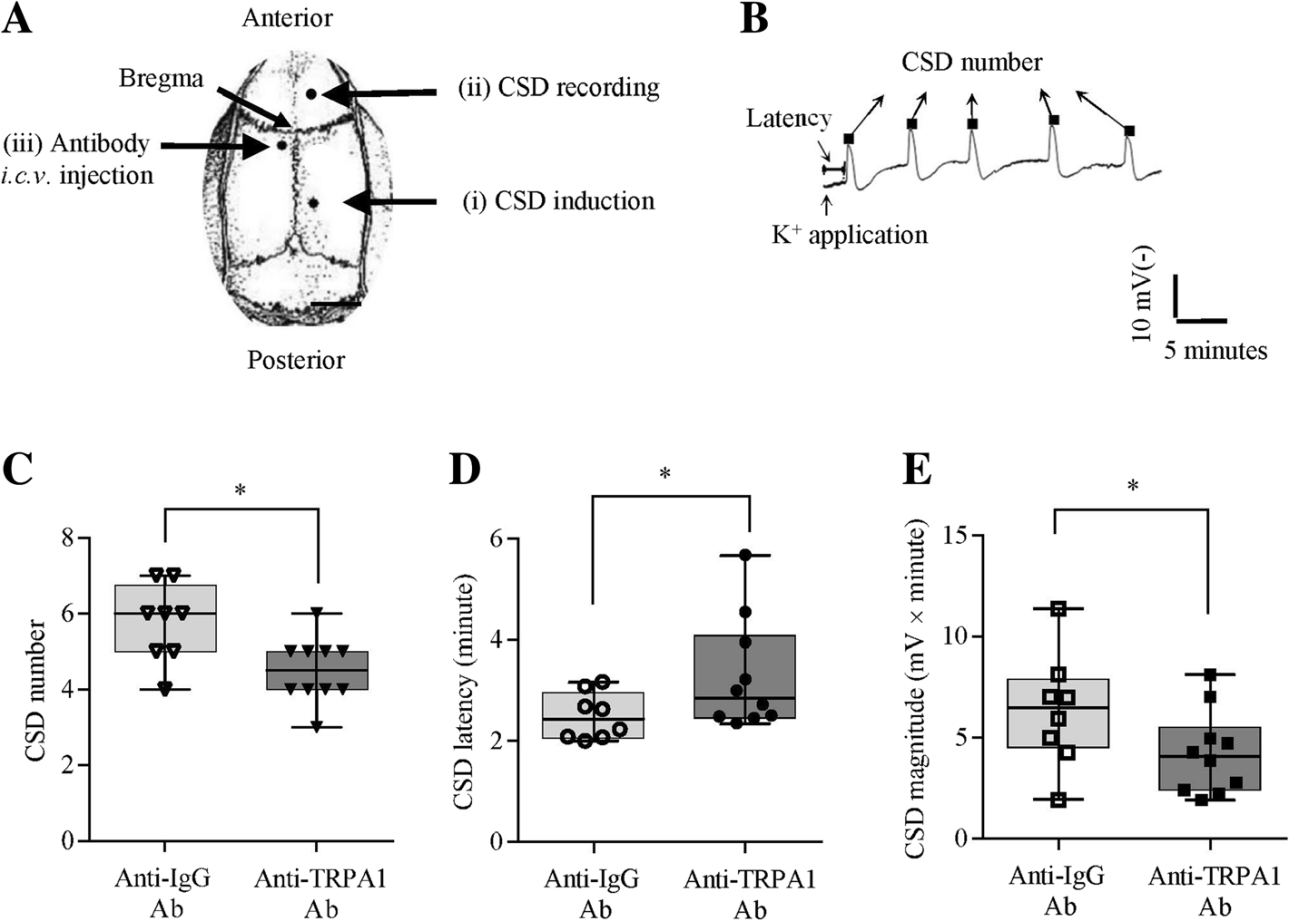
Effects of the anti-TRPA1 antibody, perfused into the contralateral ventricle, on cortical susceptibility to CSD in rats. **a** CSD was induced by topical application of 2 M KCl for 30 min onto cerebral cortex with dura intact via the posterior burr hole on the right parietal bone. The ipsilateral anterior hole was used for CSD recording. The anti-TRPA1 antibody (i, *n* = 10) or anti-IgG antibody (ii, *n* = 8) was perfused through a cannula implanted in the contralateral ventricle (*i.c.v*) at 4 days prior to CSD induction. In the sham group (iii), the anti-IgG antibody was *i.c.v* perfused in the absence of KCl application as the control (*n* = 7). The whole ipsilateral cerebral cortical tissue was subsequently used for detecting MDA level immediately after the in vivo experiment. **b** A representative trace showing CSD propagation wave after *i.c.v* perfusion of the anti-IgG antibody. CSD number, latency (minute) and magnitude (area under the curve of each CSD wave, mV × minute) were used for quantifying the excitation phase of CSD. The effects of the anti-TRPA1 antibody at 0.8 μg on CSD number are shown in panel (**c**), latency in panel (**d**) and magnitude in panel (**e**). All the values shown are median (range). *p < 0.05, Mann-Whitney U test with one-tailed calculation was used for comparison of the anti-IgG antibody and the anti-TRPA1 antibody group

Our previous study shows that TRPA1 deactivation inhibits CSD in the mouse brain slice [5]. Here we further investigated whether the TRPA1 channel antibody, perfused into the contralateral *i.c.v* could produce inhibitory effects on CSD in rats. In the anti-IgG antibody control group, topical application of 2 M KCl for 30 min typically elicited 6 [3] (in the form of median (range)) CSD waves that were identified by transient negative shifts of DC potential (Fig. 3b and c). The CSD latency was 2.4 (1.2) minutes (*n* = 8, Fig. 3d), and the CSD magnitude was 6.5 (9.5) mV × minute (*n* = 8, Fig. 3e) respectively.

In the anti-TRPA1 antibody group, 0.8 μg of the antibody perfused into the contralateral *i.c.v.* 4 days before CSD induction significantly reduced CSD number to 4.5 [3] (*n* = 10, *p* = 0.0128, Fig. 3c) when compared to the anti-IgG antibody group. Corresponding to this, CSD latency was prolonged to 2.9 (3.3) minutes (*n* = 10), which was significant when compared to the anti-IgG antibody control group (*p* = 0.0416, Fig. 3d). Conversely, CSD magnitude was reduced to 4.1 (6.2) mV × minute (*n* = 10, *p* = 0.0482, Fig. 3e).

### Reduction of cortical susceptibility to CSD by anti-TRPA1 antibody correlated with a lower MDA level

**Fig. 4:**
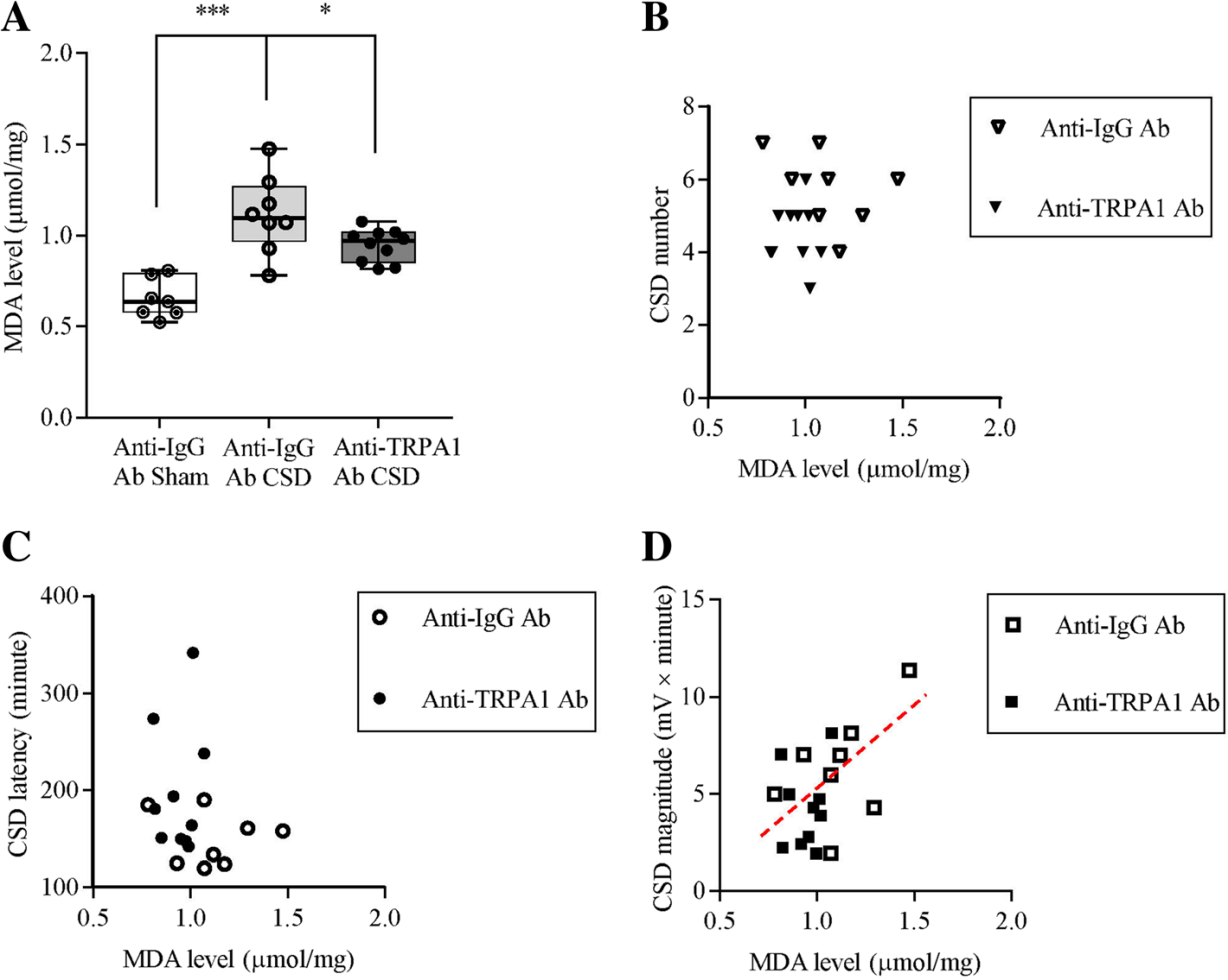
Effects of the anti-TRPA1 antibody on MDA level (μmol/mg protein) induced by CSD in the ipsilateral cerebral cortex of rat and correlation analysis of each CSD characteristic with levels of cortical ipsilateral MDA between the anti-TRPA1 antibody or anti-IgG antibody groups. **a** CSD promoted ipsilateral cortical MDA level, which was inhibited by the pretreatment of anti-TRPA1 antibody perfused into the contralateral *i.c.v* in rats. Mann-Whitney U test, one-tailed, for significance between each group (*p < 0.05, ***p < 0.001). The reduced CSD magnitude (**d**), but not CSD number (**b**) and latency (**c**) positively correlated with a lower MDA level after the anti-TRPA1 antibody perfusion. Red dotted lines indicated positive correlation between CSD magnitude and MDA level

In the anti-IgG antibody sham group, the MDA level in the ipsilateral cerebral cortex was 0.64 (0.29) μmol/mg (*n* = 7, Fig. 4a). Significant increase in MDA level was observed immediately after CSD induction and the MDA level reached to 1.07 (0.7) μmol/mg in the ipsilateral cerebral cortices of rats (*n* = 8, *p* = 0.0006, Fig. 4a), suggesting that CSD induces lipid peroxidation. As expected, 0.8 μg anti-TRPA1 antibody perfused into contralateral *i.c.v.* partially prevented the elevation of MDA level induced by CSD (*n* = 10). This reduction was significant from that of the CSD group (*p* = 0.0273, Fig. 4a), suggesting that the lipid peroxidation induced by CSD could be reduced by TRPA1 deactivation.

Correlation analysis of CSD characteristics with levels of rat ipsilateral cerebral cortical MDA in the anti-IgG and anti-TRPA1 antibodies groups showed that after perfusion of the anti-TRPA1 antibody into the *i.c.v* prior to CSD induction, the lower level of MDA showed a positive correlation with the reduced CSD magnitude (*p* = 0.0293, r = 0.5135, Fig. 4d), whilst no correlation with the reduced CSD number (Fig. 4b) and the prolonged CSD latency (Fig. 4c) was observed.

### ROS facilitated submaximal CSD induction in the mouse brain slice

**Fig. 5:**
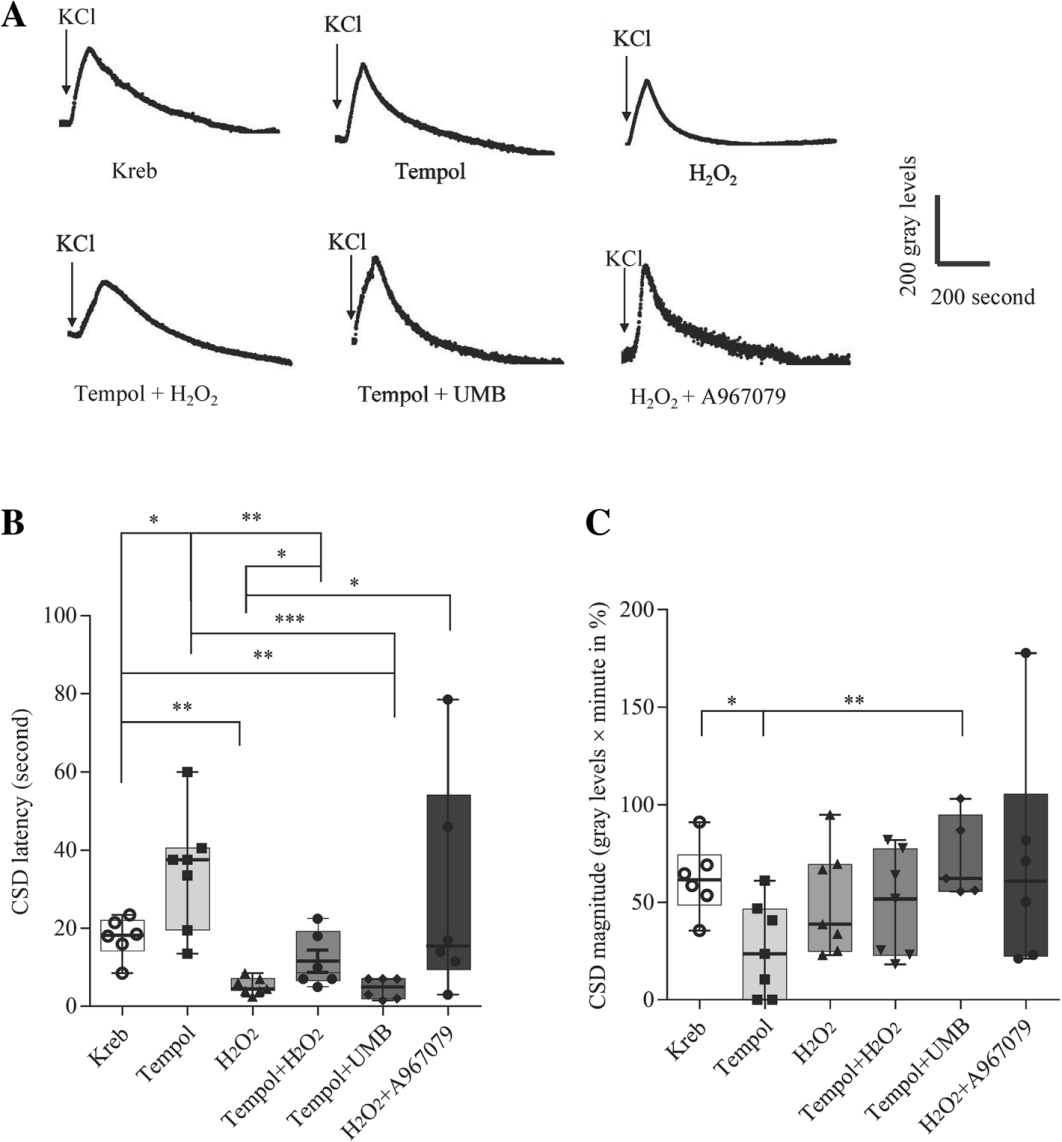
Effects of modulation of ROS and TRPA1 on cortical susceptibility to CSD and their interaction during CSD in the mouse brain slice. Submaximal CSD was induced by KCl at 50 mM. There were six groups: Kreb’s (i, *n* = 6) as control, 3 mM of the ROS inhibitor, tempol (ii, *n* = 7), 50 μM of ROS activator, H_2_O_2_ (iii, *n* = 6), 3 mM of tempol with 50 μM of H_2_O_2_ (vi, *n* = 8) or with 15 μM of the TRPA1 agonist, UMB (v, *n* = 7), 50 μM of H_2_O_2_ with 1 μM of the TRPA1 antagonist, A967079 (vi, *n* = 6). In order to minimize the animal use, data in Kreb’s control group were adopted and transformed from that in Fig. 4 in our previous paper [5]. Representative trace of the 2nd CSD episode in each group were shown in the panel **a**. The data showed that cortical susceptibility to CSD is suppressed by tempol, facilitated by H_2_O_2_, and there is a bidirectional interaction between ROS and TRPA1 in modulating latency (**b**) and magnitude (**c**) of CSD. CSD latency (second) was given as median (rang). CSD magnitude were plotted as percentage of their initial levels (1st CSD episode) and indicated as median (range). Mann-Whitney U test (one-tailed) was for significant analysis between two independent groups. * *p* < 0.05, ** *p* < 0.01, ***p < 0.001 indicate significance

We investigated whether ROS could facilitate submaximal CSD induced by 50 mM KCl in the mouse cortical brain slice. In the Kreb’s control group (*n* = 6), CSD latency was 18.3 [15] seconds in the 2nd CSD episode and CSD magnitude was 61.5% (55.48%) relative to that in the 1st CSD episode (Fig. 5b, c). The ROS inhibitor, tempol, at 3 mM (*n* = 7) significantly prolonged the latency of submaximal CSD in the 2nd episode with a 2.0-fold increase relative to that of the Kreb’s group (*p* = 0.0239, Fig. 5b); whilst CSD magnitude was reduced to 26.7% relative to its baseline (*p* = 0.0111, Fig. 5c). Conversely, pre-incubation of the ROS activator, H_2_O_2_, at 50 μM (*n* = 7) shortened the submaximal CSD latency compared to that of the Kreb’s group (*p* = 0.0012, Fig. 5b). Corresponding to this, the shortening of submaximal CSD latency by H_2_O_2_ was reversed by its co-application with tempol and significant difference was observed when compared with tempol alone group (*n* = 7, *p* = 0.0041) and the H_2_O_2_ alone group (*p =* 0.0152, Fig. 5b) respectively. These data suggest a key role of ROS in facilitating CSD induction. It was noted that there was no significant difference in the magnitude of submaximal CSD between co-application of H_2_O_2_ and tempol when compared with tempol and H_2_O_2_ group alone respectively (Fig. 5c).

### Bidirectional interplay between TRPA1 and ROS in regulating CSD

Our previous study shows that activation of TRPA1 by UMB is capable of facilitating submaximal CSD induction [5]. Here, we examined the functional interplay between ROS and TRPA1 in regulating submaximal CSD induction in the mouse brain slice. We observed that both the prolonged latency of submaximal CSD and the reduced magnitude by the antioxidant, tempol, application at 3 mM was markedly reversed in the presence of the TRPA1 activator, UMB at 15 μM (*n* = 6, Fig. 5). There was significant difference in both CSD latency (*p* = 0.0006, Fig. 5b) and magnitude (*p* = 0.0051, Fig. 5c) when compared with the tempol alone group, suggesting TRPA1 activation modulates ROS-mediated CSD. Consistently, the shortening of the submaximal CSD latency by H_2_O_2_ at 50 μM was also reversed by the TRPA1 inhibitor, A967079 at 1 μM (*n* = 6, *p* = 0.0163, Fig. 5b). Taken together, these data suggest a bidirectional link between TRPA1 activation and ROS in regulating cortical susceptibility to CSD. It was noted that no significant difference in CSD magnitude was observed between H_2_O_2_ at 50 μM in the absence and presence of A967079 at 1 μM (*n* = 6, Fig. 5c).

### Both ROS and TRPA1 activation reversed the reduced cortical susceptibility to CSD by the anti-CGRP antibody

**Fig. 6:**
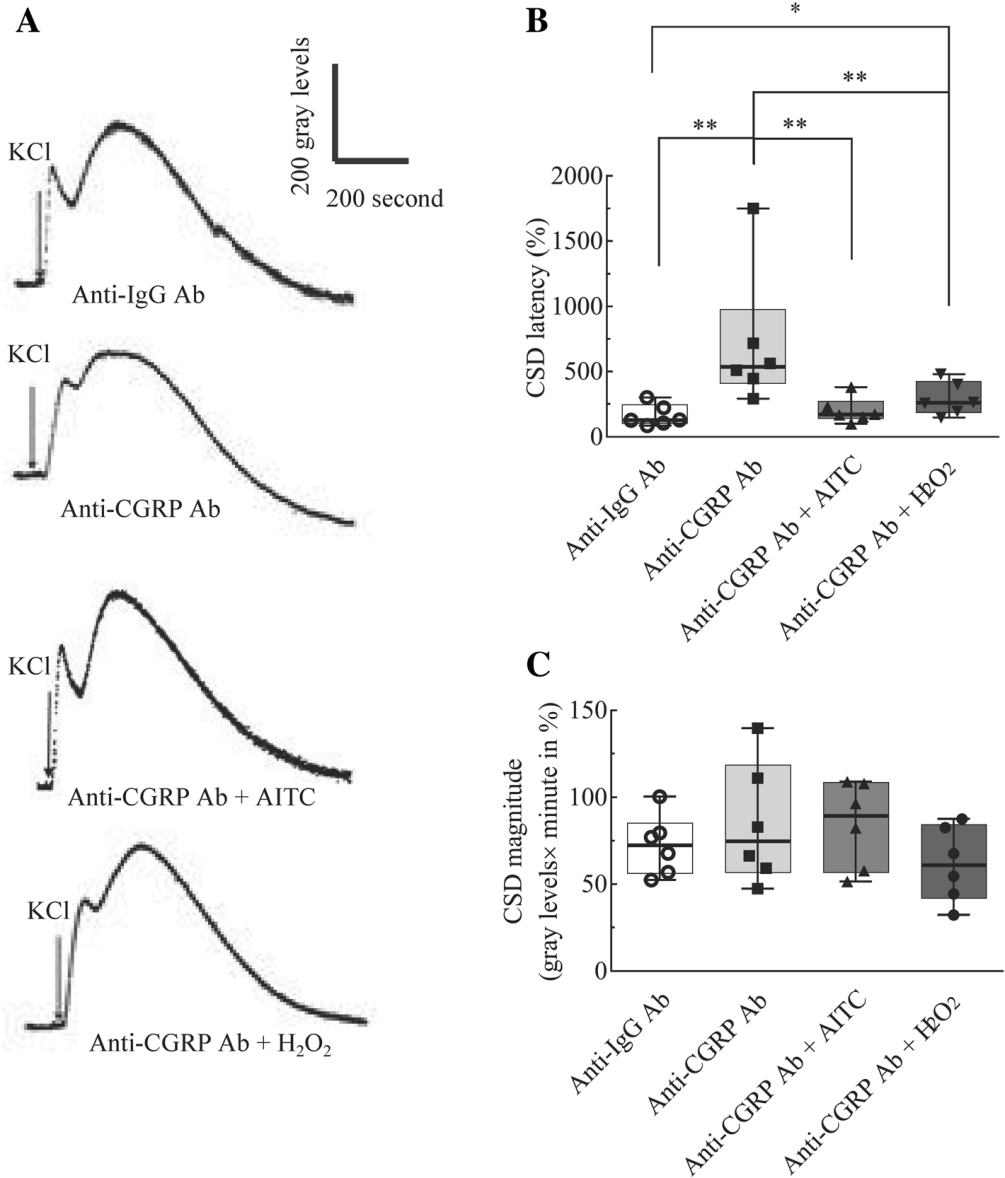
Both ROS and the TRPA1 activation reversed the inhibitory effects of the anti-CGRP antibody on CSD in the mouse brain slice. CSD was induced by 260 mM KCl. There were four groups: anti-IgG antibody at 0.025 μM (i, *n* = 6) as the control, anti-CGRP antibody at 0.4 μM in the absence (ii, *n* = 6) or presence of 50 μM of the TRPA1 agonist, AITC (iii, *n* = 6) or the ROS activator, H_2_O_2_ (vi, *n* = 6). In order to minimize the animal use, data in anti-IgG antibody control group and the anti-CGRP antibody were adopted and transformed from that in Fig. 5 in our recent paper [5]. Representative trace of the 2nd CSD episode in each group are shown in the panel **a**. The data showed that both ROS and the TRPA1 activation reversed the prolonged CSD latency (**b**), but not magnitude (**c**) under the perfusion of the anti-CGRP antibody. Data were plotted as percentage of their initial levels (1st CSD episode) and indicated as median (range). Mann-Whitney U test, one-tailed, was used for significant analysis between two independent groups. *p < 0.05, **p < 0.01. Abbreviation: Ab indicates antibody

We further examined whether ROS and TRPA1 activation would produce similar effects on preventing the reduced cortical susceptibility to CSD by inhibition of CGRP, a known target for migraine prevention. As reported previously [5], the anti-CGRP antibody incubated at 0.4 μM markedly prolonged CSD latency (*n* = 6, *p* = 0.0022, Fig. 6b) when compared with the anti-IgG antibody group (*n* = 6). H_2_O_2_ at 50 μM markedly shortened the prolonged CSD latency, which was significant when compared to the anti-CGRP antibody alone group (*n* = 6, *p* = 0.0076, Fig. 6b). The CSD latency did not reverse to basal level as there was a significant difference (*n* = 6, *p* = 0.0325, Fig. 6b). Similar as the ROS activator, the TRPA1 activator, AITC at 50 μM, also significantly reversed the prolonged CSD latency by the anti-CGRP antibody (*n* = 6, *p* = 0.0022, Fig. 6b). The anti-CGRP antibody did not alter CSD magnitude and neither H_2_O_2_ nor AITC altered CSD magnitude (Fig. 6c).

## Discussion

In this study, we demonstrate that TRPA1 deactivation reduces the likelihood for CSD induction in rats, which coincides with the reduction of ipsilateral cortical lipid peroxidation promoted by CSD. We also show that the ROS-facilitated submaximal CSD induction can be reversed by deactivation of TRPA1 in the mouse brain slice; whereas, the reduced cortical susceptibility to CSD by ROS inhibition in return is reversed by TRPA1 activation. Furthermore, both ROS and TRPA1 activation are capable of reversing the prolonged CSD latency under CGRP inhibition in the mouse brain slice. Our data reveal that ROS/TRPA1/CGRP signaling contributes to CSD induction. We propose that there is a positive feedback loop among these signals by which TRPA1 exerts in stress-associated migraine.

A key finding of the present study is that TRPA1 deactivation reduces the likelihood for CSD induction in rats since pre-treatment of the anti-TRPA1 antibody into the contralateral *i.c.v* prolonged CSD latency and reduced CSD number and magnitude (Fig. 3). These data are compatible with our previous in vitro study that deactivation of TRPA1 by both the anti-TRPA1 antibody and TRPA1 antagonists reduces cortical susceptibility to CSD [11], whilst TRPA1 activation facilitates the propagation of submaximal CSD in the mouse brain slice [5]. Our study suggest that TRPA1 plays an important role in migraine pathogenesis through central mechanism. Given that TRPA1-deficient mice lack allodynia [21] and TRPA1 channel activation facilitates the development of neuropathic pain [25], Collectively, anti-TRPA1 therapies are likely to form a preventive strategy for migraine prophylaxis.

It is possible that the action of TRPA1 on CSD in rats involves both neurons and glial cells for the following reasons. First, the inhibitory role in CSD under TRPA1 deactivation should derivate from the central but not the peripheral as the anti-TRPA1 antibody perfused into *i.c.v*, can diffuse to the cortical regions. Second, TRPA1-mediated spontaneous Ca^2+^ influx increase modulates the spontaneous release of peptide hormones from rat primary astrocytes [26]. Third, the role of TRPA1 on CSD is seen in the mouse brain slice where TPRA1 located in the cerebral cortex rather than indirectly through peripheral TRPA1 [5]. Further, the fact that TRPA1 is expressed in neurons, and to a lesser extent, glial cells in cerebral cortices of rats (Fig. 1) and mice (Fig. 2) support their neuronal and/or glial functions during CSD. Nevertheless, we cannot rule out a vascular role of TRPA1 in CSD and this requires future investigation.

CSD induces ROS production in the trigeminal nociceptive system [10]. Here, we show that CSD is capable of promoting cerebral cortical lipid peroxidation in rats (Fig. 4a). The importance of ROS in CSD is supported by that exogenous ROS by H2O2 facilitates submaximal CSD induction in the mouse brain slice and these effects can be reversed by the antioxidant (Fig. 5). These data are consistent with that ROS inhibition by the antioxidant reduces the likelihood for CSD occurrence in vivo [11]. Taken together, we conclude that ROS play crucial roles in CSD elicitation and propagation. These data also support that CSD is an important model of stress-related migraine since oxidative stress is generated by all common migraine triggers [7].

It is known that TRPA1 is sensitive to oxidative stress. How does TRPA1 interact with ROS during CSD? Here we show that ROS is involved in TRPA1 signaling in modulating CSD as deactivation of TRPA1 channel is able to reduce cortical susceptibility to CSD, which coincides with a lower level of ROS (Fig. 4d). It should be noted that no correlation with the reduced CSD number (Fig. 4b) and the increase in CSD latency (Fig. 4c) was seen relative to control. The reason to account for the correlation difference with different CSD parameters are not known, which requires further investigation. Interestingly, our data show a bidirectional interaction between ROS and TRPA1 in mediating cortical susceptibility to CSD in the mouse brain slice (Fig. 5). On one side, facilitation of submaximal CSD induction by exogenous ROS, H_2_O_2,_ can be reversed under TRPA1 antagonism by A-967079 (Fig. 5), suggesting ROS regulates TRPA1 activity in mediating CSD. On the other side, TRPA1 activation in return reverses the prolonged CSD latency and reduced CSD magnitude by ROS inhibition (Fig. 5). These data are compatible with reports that degradation of H_2_O_2_ prevents NADPH-induced TRPA1 activation in cerebral endothelium of mice [27]. It is reasonable to propose that ROS is required for TRPA1 signaling in migraine progression.

Another finding of this study is that TRPA1/CGRP signaling plays a critical role in regulating cortical susceptibility to CSD. First, the reduced cortical susceptibility is observed under both TRPA1 deactivation (Fig. 3) and CGRP antagonism (Fig. 6) as that was reported previously [5]. Second, the involvement of CGRP in cortical TRPA1 signaling in CSD was supported by that exogenous CGRP abrogates the suppressive effect of TRPA1 antagonism on CSD in the mouse brain slice [5]; whereas TRPA1 activation in return reverses the prolonged CSD latency under CGRP antagonism (Fig. 6b). These data suggest that TRPA1 and CGRP have bidirectional interaction in regulating cortical susceptibility to CSD. As of TRPA1, the role of CGRP during CSD observed in this study is also from central since the mouse brain slice was used. Yet, how TRPA1 and CGRP mutually interact in regulating cortical susceptibility to CSD requires further study.

**Fig. 7:**
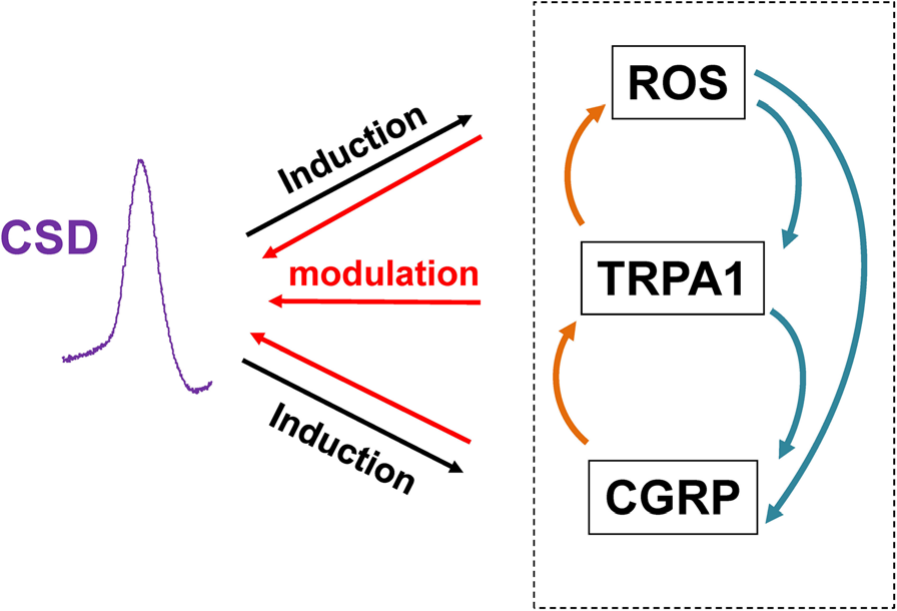
A diagram depicting ROS/TRPA1/CGRP signaling in modulating cortical susceptibility to CSD. It is proposed that ROS may trigger TRPA1 activation and CGRP production, which create a positive feedback loop in regulating cortical susceptibility to CSD. In which way, ROS could facilitate CSD propagation for the subsequent development of migraine

We found, similar as TRPA1 activation, the ROS activator also reverses the prolonged CSD latency by the anti-CGRP antibody (Fig. 6b). It is likely that ROS may facilitate CGRP production, which enables ROS being capable of reversing the reduced cortical susceptibility to CSD after CGRP inhibition (Fig. 6). This is supported by that ROS facilitates CGRP production in trigeminal nociceptive system [10] and that multiple CSD induces both ROS production in rat cortices (Fig. 4a) and CGRP production in both brain slices and cerebral cortices of rats [6, 28]. Given that there is an interaction among ROS, TRPA1 and CGRP in mediating CSD as discussed above and inhibition of TRPA1 blocks ROS-induced CGRP release [29], we propose that ROS triggers both TRPA1 activation and CGRP production, which create a positive feedback loop in regulating cortical susceptibility to CSD (Fig. 7). In which way, ROS could facilitate CSD propagation for the subsequent development of migraine. This proposal is in line with reports that TRPA1 activation by environmental irritants stimulates CGRP release in rat trigeminal ganglia neurons [29] and inhibition of TRPA1 activation blocks ROS-induced CGRP release [10]. However, whether deactivation of TRPA1 channel prior to CSD induction would subsequently regulate CGRP production remains further investigation.

## Conclusion

ROS/TRPA1/CGRP signaling plays a critical role in regulating cortical susceptibility to CSD, which is of central mechanism. We propose that there is a positive feedback loop among these signals by which TRPA1 exerts in stress-associated migraine. Anti-CGRP therapies have brought great hopes for effective prevention of episodic and chronic migraine, yet there are still non-responders to these therapies [4]. Our data suggest that reduction of ROS production and blockade of TRPA1 may provide alternative therapeutic benefits to CGRP in preventing migraine. These signals may also have clinical implications to traumatic brain injury as CSD also occurs following acute brain injuries and strokes [30, 31].
